# Comparison of the Effectiveness of Prolonged Infusion and Intermittent Infusion of Meropenem in Patients With Sepsis: A Meta-Analysis

**DOI:** 10.7759/cureus.46990

**Published:** 2023-10-13

**Authors:** Adnan Lokhandwala, Premalkumar Patel, Abraham K Isaak, Rao Faizan Yousaf, Abdalkareem Nael Jameel Maslamani, Sondos K Khalil, Eman Riaz, Shamsha Hirani

**Affiliations:** 1 Medicine, Khalifa University, Abu Dhabi, ARE; 2 Infectious Disease, Mount Sinai Medical Center, Miami Beach, USA; 3 Telemetry, Sharp Memorial Hospital, San Diego, USA; 4 Internal Medicine, Orotta School of Medicine and Dentistry, San Diego, ERI; 5 Internal Medicine, Faisalabad Medical University, Faisalabad, PAK; 6 General Physician, Cairo University, Cairo, EGY; 7 Internal Medicine, Hamad Medical Corporation, Doha, QAT; 8 Internal Medicine, Chiniot General Hospital, Karachi, PAK; 9 Cardiology, Baqai Hospital, Karachi, PAK

**Keywords:** systematic analysis and meta-analysis, sepsis, meropenem, intermittent infusion, prolonged infusion

## Abstract

The aim of this study was to compare the clinical effectiveness of prolonged infusion and intermittent infusion of meropenem in patients with sepsis. This meta-analysis was conducted following the Preferred Reporting Items for Systematic Reviews and Meta-Analysis (PRISMA) 2020 guidelines. PubMed, Web of Science, Scopus, and the Cochrane Library were searched without any language or time restrictions, up to September 25, 2023. The primary outcomes assessed in this meta-analysis included clinical success and all-cause mortality. Other outcomes assessed in this study encompassed the mean length of ICU stay. Total eight studies met the eligibility criteria and were included in this meta-analysis. Pooled analysis showed that the clinical success rate was significantly higher in patients receiving prolonged infusion of meropenem compared to intermittent infusion (RR: 1.49, 95% CI: 1.30 to 1.70). All-cause mortality was 24% significantly lower in patients receiving prolonged infusion of meropenem compared to intermittent infusion (RR: 0.76, 95% CI: 0.60 to 0.96). The results suggest that prolonged infusion of meropenem could be a more effective and efficient treatment for sepsis patients. However, more randomized controlled trials are needed to confirm these findings and to establish the optimal dosing and administration schedule for prolonged infusion of meropenem.

## Introduction and background

Sepsis remains a significant cause of illness and death among patients in the intensive care unit (ICU) [1]. Commencing effective antimicrobial treatment early is a crucial aspect of managing severe sepsis and septic shock [2]. The ICU faces a substantial challenge due to the rising resistance of bacteria to antibiotics, which has led to slower and costlier development of suitable antibacterial drugs [3]. It is imperative to use antibiotics at optimal doses and via appropriate routes to enhance their effectiveness and reduce the risk of bacteria developing resistance [4].

β-Lactam antibiotics are the most commonly prescribed intravenous antimicrobials in the US, constituting over 65% of such prescriptions [5]. These antibiotics, which include Meropenem, are time-dependent, meaning their effectiveness depends on the duration they maintain a concentration above the minimal inhibitory concentration (MIC) [6]. For carbapenems like Meropenem, a minimum of 40% of the dosing interval (referred to as % of T>MIC) is needed for effectiveness [6], with clinical and bacteriological outcomes being significantly better when T>MIC reaches 100% in patients with serious bacterial infections [7].

Pharmacokinetic investigations conducted in both non-critically ill and critically ill patients have shown that the consistent achievement of drug levels associated with maximum antibacterial effectiveness is possible when β-lactam antibiotics are administered through continuous infusion (CI) [8,9]. There continues to be an ongoing debate regarding whether traditional intermittent bolus (IB) dosing or CI is the preferred clinical approach for administering beta-lactam antibiotics [10]. Despite this debate, pharmacodynamic (PD) data on beta-lactam antibiotics indicates several advantages for CI, as it operates on a time-dependent mechanism, with the duration of time (T) during which the free drug concentration remains above the minimum inhibitory concentration (MIC; fT>MIC) being the best descriptor of its ability to kill bacteria [11]. Consequently, CI offers potential benefits by consistently maintaining higher antibiotic concentrations above the MIC. Consequently, the CI of meropenem has been proposed as a means to maximize therapeutic potential in critically ill patients. Recent trials have explored the use of continuous meropenem administration in sepsis patients, demonstrating improved pharmacokinetic efficacy, bacterial eradication, and clinical cure rates. However, the findings from these clinical studies are not uniform. Therefore, our objective is to assess the clinical effectiveness of prolonged infusion versus intermittent infusion of meropenem in patients with sepsis through this meta-analysis.

## Review

Methodology

This meta-analysis was conducted following the Preferred Reporting Items for Systematic Reviews and Meta-Analysis (PRISMA) 2020 guidelines.

Literature Search

PubMed, Web of Science, Scopus, and the Cochrane Library were searched without any language or time restrictions, up to September 25, 2023. Additionally, the Google Scholar search engine was manually searched to identify additional studies. Keywords used for the search included: Meropenem AND prolonged infusion OR extended infusion OR CI AND Intermittent infusion AND sepsis OR critically ill patients. Furthermore, medical subject heading (MeSH) terms were employed to enhance the search. Moreover, the reference lists of all included articles were manually screened to identify additional studies relevant to the study topic.

Study Selection

The systematic search produced a set of identified studies, which were then imported into EndNote 20 software. Duplicate entries were removed. Two researchers initially screened each publication's title and abstract based on predefined inclusion criteria. Subsequently, these same two researchers independently evaluated the full texts of studies that met the initial screening criteria. Any disagreements were resolved through discussion or consultation with a third researcher. We included randomized controlled trials (RCTs) and cohort studies (both prospective and retrospective) that compared prolonged and CI of meropenem in critically ill patients. We excluded studies that involved other β-lactam antibiotics and those comparing prolonged and intermittent infusion of drugs in the pediatric population. Additionally, case reports, case series, reviews, and editorials were excluded.

Data Extraction

Two researchers independently conducted data extraction using a uniform data extraction sheet in Microsoft Office Excel. The following data were extracted: study characteristics, including title, first author's name, publication year, country of study, sample size, and patients' characteristics, such as mean age, the number of males (n), and comorbidities. The primary outcomes assessed in this meta-analysis included the clinical success and all-cause mortality. Other outcomes assessed in this study encompassed the mean length of ICU stay.

Quality Assessment

Quality assessment is a critical component of conducting a meta-analysis to ensure the reliability and validity of the included studies. In this study, the Cochrane Risk of Bias assessment tool was employed to evaluate the quality of RCTs. This tool assesses the risk of bias in various domains, such as randomization, allocation concealment, blinding, and outcome reporting, providing a comprehensive evaluation of the methodological rigor of RCTs. For observational studies, the Newcastle-Ottawa Scale was utilized to assess quality. This scale examines observational studies in terms of the selection of study groups, comparability of groups, and assessment of outcome or exposure, offering a systematic approach to evaluating the quality and risk of bias in non-randomized studies.

Data Analysis

Data analysis was performed using RevMan Version 5.4.1. We utilized risk ratios (RR) and 95% confidence intervals (CI) to compare categorical outcomes between prolonged and intermittent meropenem infusion, and for continuous variables, we reported the mean difference (MD) along with a 95% CI. To account for variations among the study results, we employed random-effect models, regardless of the level of heterogeneity. A significance level was set at a p-value of 0.05. Heterogeneity among the study results was assessed using the I-square statistic, with an I-square value exceeding 50% indicating significant heterogeneity.

Results

**Figure 1:**
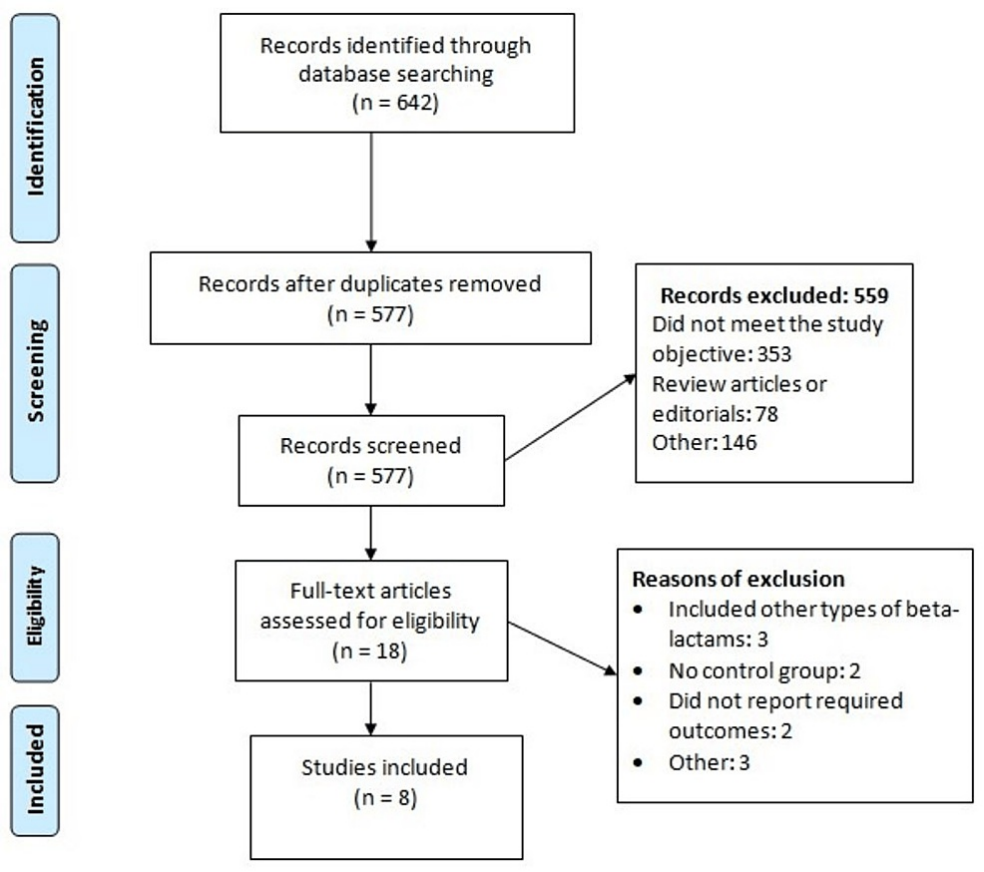
PRISMA flowchart of selection of studies

**Figure 2:**
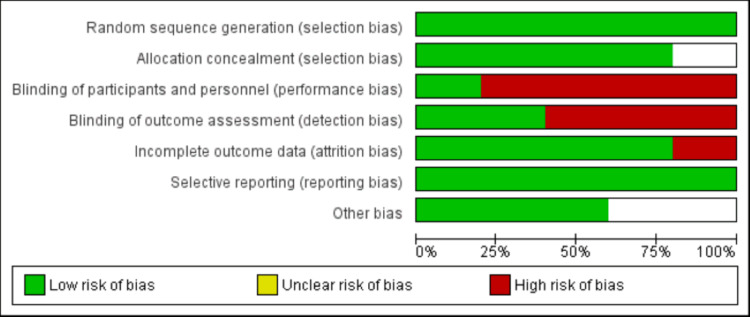
Risk of bias graph of included RCTs

In the first step, 642 articles were identified, of which 65 were duplicates and removed. After screening the abstracts and titles of the remaining 577 reports, another 559 reports were excluded and remaining 18 articles being further assessed for eligibility criteria. Finally, eight studies met the eligibility criteria and were included in our meta-analysis. Figure 1 shows the PRISMA flowchart of study selection. Table 1 presents the characteristics of included studies. Five studies were RCTs. Figure 2 presents risk of bias assessment of included RCTs. Table 2 shows quality assessment of observational studies.

**Table 1:** Characteristics of included studies RCT: Randomized-control trial; NR: Not reported

Author Name	Year	Study Design	Region	Groups	Sample Size	Mean Age (Years)	Male (n)
Ahmed et al. [12]	2020	Observational	United States	Prolonged	52	68	26
				Intermittent	96	68	56
Chytra et al. [13]	2012	RCT	Czech Republic	Prolonged	120	44.9	78
				Intermittent	120	47.2	83
Feher et al. [14]	2014	Observational	Spain	Prolonged	76	44	44
				Intermittent	88	49.5	42
Helmy et al. [15]	2015	RCT	Egypt	Prolonged	50	NR	NR
				Intermittent	50		
Lorente et al. [16]	2006	Observational	Spain	Prolonged	42	57.25	33
				Intermittent	47	56.46	38
Monti et al. [17]	2023	RCT	Multi-national	Prolonged	303	65.5	195
				Intermittent	304	63.4	209
Wang et al. [18]	2014	RCT	China	Prolonged	38	NR	NR
				Intermittent	40		
Zhao et al. [19]	2017	RCT	China	Prolonged	25	68	10
				Intermittent	25	67	11

**Table 2:** Quality assessment of observational studies

Study ID	Selection	Comparability	Outcome	Overall
Ahmed et al. [12]	3	2	3	8
Feher et al. [14]	3	1	3	7
Lorente et al. [16]	3	2	3	8

Clinical Success Rate

**Figure 3:**
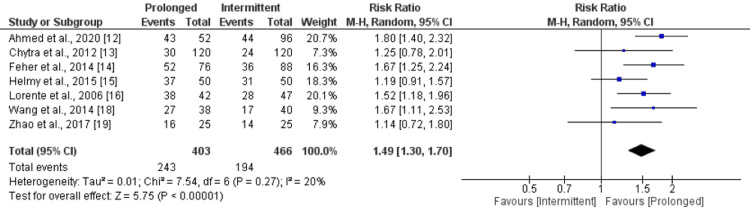
Comparison of clinical success rate References: [12-16,18,19]

Seven studies compared the clinical success rate between prolonged and intermittent infusion of meropenem. As shown in Figure 3, clinical success rate was significantly higher in patients receiving prolonged infusion of meropenem compared to intermittent infusion (RR: 1.49, 95% CI: 1.30 to 1.70). Low heterogeneity was reported among the study results (I-square: 20%).

All-Cause Mortality

**Figure 4:**
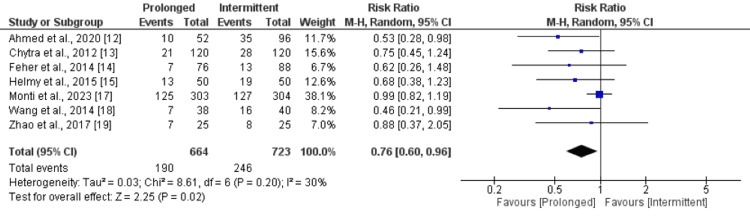
Comparison of all-cause mortality References: [12-15,17-19]

Seven studies were included in the pooled analysis of the comparison of all-cause mortality between prolonged and intermittent infusion of meropenem. As shown in Figure 4, all-cause mortality was 24% significantly lower in patients receiving prolonged infusion of meropenem compared to intermittent infusion (RR: 0.76, 95% CI: 0.60 to 0.96). Low heterogeneity was reported among the study results (I-square: 30%).

Duration of Intensive Care Unit (ICU) Stay in Days

**Figure 5:**
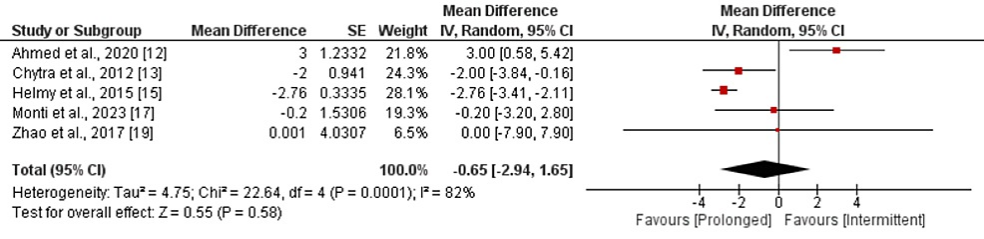
Comparison of ICU stay References: [12,13,15,17,19]

Seven studies were included in the pooled analysis of comparison of ICU LOS between two groups. As shown in Figure 5, overall LOS was not significantly different between two groups (MD: -0.65, 95% CI: -2.94 to 1.65). High heterogeneity was reported among the study results (I-square: 82%). Due to high heterogeneity, we performed sensitivity analysis by removing the study conducted by Ahmed et al. After removing this study, we found that LOS in ICU was significantly lower in patients receiving prolonged infusion of meropenem compared to intermittent infusion (MD: -2.37, 95% CI: -3.26 to -1.49). I-square reduced from 82% to 14%.

Discussion

This meta-analysis was carried out to compare the treatment outcomes of patients who received meropenem through prolonged infusion versus those who received it intermittently. In our analysis, which incorporated data from eight studies involving a total of 1,476 patients, we observed that prolonged meropenem infusion was associated with better outcomes. This included a reduced mortality rate and a higher success rate when compared to intermittent meropenem infusion. The findings from this meta-analysis underscore the potential for substantial clinical improvement in the treatment of sepsis patients through the adoption of prolonged meropenem infusion. Importantly, a meta-analysis conducted by Chen et al., which explored the comparison between continuous and intermittent meropenem infusion, reported results that align with our findings [20].

There exists a wealth of research dedicated to optimizing the pharmacokinetics/pharmacodynamics (PK/PD) of beta-lactam antibiotics such as meropenem. This extensive body of research suggests that applying PK/PD principles is more likely to help critically ill patients achieve their treatment targets compared to the conventional intermittent dosing approach [21,22]. Recent studies have also delved into evaluating the practical advantages of implementing PK/PD-guided interventions through RCTs, with results indicating a potential advantage of this approach, particularly for patients with severe sepsis [23]. Several pharmacokinetic studies involving critically ill sepsis patients have reported that CI of meropenem leads to a higher likelihood of reaching target concentrations, both in plasma and in tissues, as compared to intermittent dosing [24]. Furthermore, it's worth noting that previous meta-analyses, unlike our present analysis, were less discerning in their inclusion criteria. They encompassed data from both critically ill and non-critically ill patients and allowed for the inclusion of various β-lactam antibiotics in the two treatment groups. This broader scope may have diluted the potential advantages of CI [25]. However, in our meta-analysis, we maintained a more focused approach, exclusively assessing studies involving meropenem [20].

Our results demonstrated that prolonged meropenem infusion not only improved clinical outcomes but also led to reduced ICU stays and overall hospitalization times. This suggests that continuous administration may offer a more economically efficient therapy option for sepsis patients. Similar findings were reported in studies involving comparable beta-lactam antibiotics, as evidenced by Nicasio et al. in 2010 (indicating shorter infection-related stays), Lodise et al. in 2007, and Merchant et al. in 2008 (both demonstrating shorter hospital stays) [26-28].

Several limitations should be taken into account when interpreting the findings of this meta-analysis. Firstly, the limited number of studies and participants included in this analysis diminishes the reliability of the conclusions drawn. Secondly, the presence of heterogeneity in treatment protocols and drug dosages contributes to the clinical diversity observed in the data. Thirdly, the criteria employed in the majority of trials to define and assess the severity of sepsis do not align with the current definitions. Lastly, due to lack of individual level data, we were unable to perform subgroup analysis to understand the outcomes in different groups of patients. We need more RCTs to compare the best approach of administration of meropenem to guide the clinical practice.

## Conclusions

In summary, our comprehensive meta-analysis, which included data from eight distinct studies involving a total of 1,476 sepsis patients, yielded compelling evidence in favor of prolonged infusion of meropenem as a superior treatment strategy. Notably, this approach demonstrated marked improvements in patient outcomes, showcasing a noteworthy reduction in mortality rates and a significant increase in clinical success rates when compared to the conventional intermittent infusion method. These findings underscore the potential of prolonged infusion of meropenem as a more efficacious and efficient therapeutic option for sepsis patients. Nonetheless, it is crucial to exercise caution and recognize the need for further research to solidify these conclusions. Future RCTs, with larger and more diverse patient populations, should be conducted to corroborate our results and ascertain the optimal dosing and administration regimen for prolonged meropenem infusion. These investigations will be instrumental in refining sepsis treatment protocols and ultimately enhancing patient care in the context of this life-threatening condition.
